# Poly[[tetra­aqua­tetra­kis­[μ_3_-5-(pyridine-4-carboxamido)­isophthalato]cobalt(II)dierbium(III)] tetra­hydrate]

**DOI:** 10.1107/S1600536811039948

**Published:** 2011-10-05

**Authors:** Yi-Fang Deng, Man-Sheng Chen, Chun-Hua Zhang, Xiong-Wen Tan

**Affiliations:** a Key Laboratory of Functional Organometallic Materials, Hengyang Normal University, Department of Chemistry and Materials Science, Hengyang, Hunan 421008, People’s Republic of China

## Abstract

In the centrosymmetric polymeric title compound, {[CoEr_2_(C_14_H_8_N_2_O_5_)_4_(H_2_O)_4_]·4H_2_O}_*n*_, the Er^III^ cation has a coordination number of eight and is surrounded by seven carboxyl­ate O atoms from four 5-(pyridine-4-carboxamido)­isophthalate (*L*) ligands and one water mol­ecule, forming a distorted square-anti­prismatic arrangement. The Co^II^ cation is located on an inversion center and is coordinated by two pyridine N atoms, two carboxyl­ate O atoms and two water mol­ecules in a distorted octa­hedral geometry. The asymmetric unit contains two anionic *L* ligands. One bridges two Er^III^ cations and one Co^II^ cation through two carboxyl­ate groups and one pyridine N atom, while the other bridges two Er^III^ cations and one Co^II^ cation through two carboxyl­ate groups. Extensive O—H⋯O, O—H⋯N and N—H⋯O hydrogen-bonding inter­actions are present in the crystal, involving all uncoordinated water mol­ecules and the uncoordinated pyridine N atom of one of the ligands bonded to an adjacent coordinated water mol­ecule. The title compound is isotypic with the gadolinium analogue.

## Related literature

For the isotypic structure of the gadolinium analogue, see: Deng *et al.* (2011 ▶). For related hetero-metallic complexes, see: Chen *et al.* (2011*a* ▶,*b* ▶); Gu & Xue (2006 ▶); Liang *et al.* (2000 ▶); Prasad *et al.* (2007 ▶); Zhao *et al.* (2003 ▶, 2004 ▶).
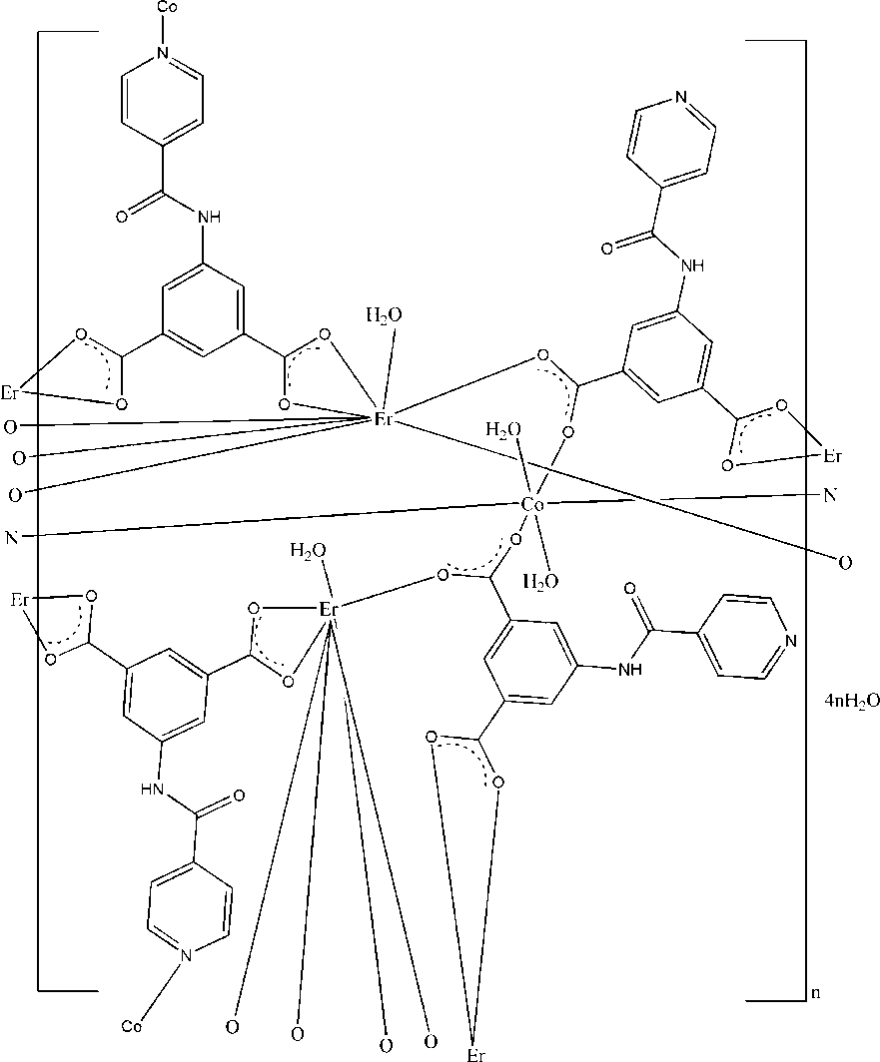


## Experimental

### 

#### Crystal data

[CoEr_2_(C_14_H_8_N_2_O_5_)_4_(H_2_O)_4_]·4H_2_O
*M* *_r_* = 1674.47
Triclinic, 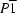

*a* = 10.0816 (9) Å
*b* = 10.7844 (10) Å
*c* = 13.7316 (12) Å
α = 79.174 (1)°
β = 78.771 (2)°
γ = 86.355 (2)°
*V* = 1437.7 (2) Å^3^
*Z* = 1
Mo *K*α radiation
μ = 3.28 mm^−1^
*T* = 293 K
0.20 × 0.18 × 0.10 mm

#### Data collection

Bruker SMART CCD area-detector diffractometer
Absorption correction: multi-scan (*SADABS*; Bruker, 2000 ▶) *T*_min_ = 0.560, *T*_max_ = 0.735
7172 measured reflections
4979 independent reflections
4649 reflections with *I* > 2σ(*I*)
*R*_int_ = 0.096

#### Refinement

*R*[*F* ^2^ > 2σ(*F* ^2^)] = 0.052
*wR*(*F* ^2^) = 0.136
*S* = 1.02
4979 reflections
430 parameters
H-atom parameters constrained
Δρ_max_ = 3.52 e Å^−3^
Δρ_min_ = −2.99 e Å^−3^

### 

Data collection: *SMART* (Bruker, 2000 ▶); cell refinement: *SAINT* (Bruker, 2000 ▶); data reduction: *SAINT*; program(s) used to solve structure: *SHELXTL* (Sheldrick, 2008 ▶); program(s) used to refine structure: *SHELXTL*; molecular graphics: *XP* in *SHELXTL* and *DIAMOND* (Brandenburg, 1999 ▶); software used to prepare material for publication: *SHELXTL*.

## Supplementary Material

Crystal structure: contains datablock(s) global, I. DOI: 10.1107/S1600536811039948/wm2536sup1.cif

Structure factors: contains datablock(s) I. DOI: 10.1107/S1600536811039948/wm2536Isup2.hkl

Additional supplementary materials:  crystallographic information; 3D view; checkCIF report

## Figures and Tables

**Table 1:** Hydrogen-bond geometry (Å, °)

*D*—H⋯*A*	*D*—H	H⋯*A*	*D*⋯*A*	*D*—H⋯*A*
N1—H1⋯O3*W*^i^	0.86	2.16	3.007 (8)	166
O1*W*—H1*WB*⋯O4*W*^ii^	0.85	2.05	2.769 (9)	142
O1*W*—H1*WA*⋯O8^iii^	0.85	2.22	2.975 (8)	149
O2*W*—H2*WB*⋯O4*W*^iv^	0.85	2.32	3.109 (10)	156
O2*W*—H2*WC*⋯N2^v^	0.85	1.94	2.687 (8)	147
O3*W*—H3*WA*⋯O7^vi^	0.85	2.30	3.041 (8)	147
O3*W*—H3*WB*⋯O9	0.85	2.30	3.042 (8)	147
O4*W*—H4*WA*⋯O3^vii^	0.85	1.92	2.729 (8)	159
O4*W*—H4*WB*⋯O6^viii^	0.85	1.95	2.762 (8)	159

Symmetry codes: (i) 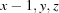; (ii) 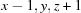; (iii) 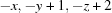; (iv) 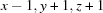; (v) 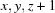; (vi) 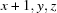; (vii) 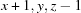; (viii) 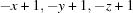.

**Fig. 1.**
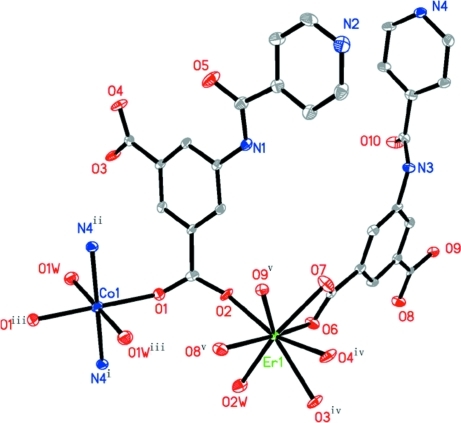
The ORTEP drawing of the title compound (I). Displacement ellipsoids are drawn at the 30% probability level. [Symmetry codes: (i) -1 + x, y, 1 + z (ii) -x, 1 - y, 1 - z (iii) -1 - x, 1 - y, 2 - z (iv) x, 1 + y, z (v)-1 + x, y, z.]

**Fig. 2.**
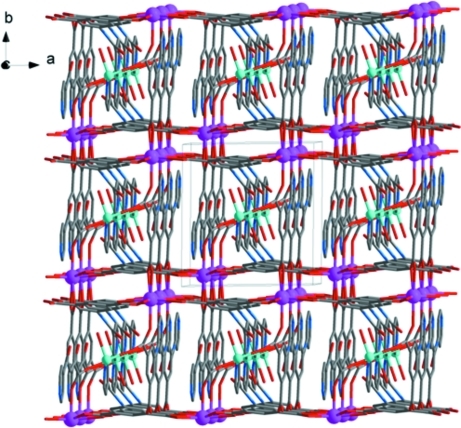
Projectionn along [001] showing the three-dimensional structure of the compound. Colour code: Er (pink), Co (light blue), O (red), N (blue), C (grey).

## supplementary crystallographic information

### Comment

The rational design and synthesis of higher-dimensional transition-lanthanide metal heterometallic networks have attracted increasing attention, which is justified not only by the fascinating structural diversity of the resulting architectures but also by the potential applications of these complexes as important functional solid materials (Chen *et al.*, 2011*a*,*b*; Gu & Xue, 2006; Liang *et al.*, 2000; Prasad *et al.*, 2007; Zhao *et al.*, 2003, 2004). In this context, we have synthesized a new 3*d*-4*f* coordination polymer, [CoEr_2_(C_14_H_8_O_5_N_2_)_4_(H_2_O)_4_]^.^4H_2_O, or [CoEr_2_(*L*)_4_(H_2_O)_4_]^.^4H_2_O and report its crystal structure here.

The title compound is isotypic with the gadolinium analogue (Deng *et al.*, 2011). The central Er^III^ ion is eight-coordinated by seven O atoms from four *L*^2-^ ligands and one water molecule, forming a distorted square antiprismatic arrangement around the metal (Fig. 1). The carboxyl groups of the two unique *L*^2-^ ligands exhibit different coordination modes: one coordinates to two Er^III^ and one Co^II^ atom (site symmetry 1) using its two carboxylate groups with bidentate-chelate and bis-monodentate coordination modes while the pyridyl group is free of coordination. The other ligand coordinates to two Er^III^ ions through the carboxylate groups with a bidentate-chelate coordination mode and to one Co^II^*via* the pyridyl group. Based on the coordination modes of the carboxylate and pyridyl groups of the ligands, a three-dimensional network is formed (Fig. 2), which is similar to that of complexes of the type {[*Ln*Co_0.5_(INAIP)_2_(H_2_O)_2_].2H_2_O}_n_ (Chen, *et al.* 2011*b*). Extensive O—H···O and N—H···O hydrogen bonding interactions are present in the crystal structure (Table 1).

### Experimental

A mixture of 0.05 mmol Er(NO_3_)_3_^.^6H_2_O (22.5 mg. 0.05 mmol), H_2_*L* (28.6 mg, 0.1 mmol), Co(OAc)_2_.4H_2_O (13.2 mg, 0.05 mmol), NaOH (6.0 mg, 0.15 mmol), MeOH (4 ml) and H_2_O (6 ml) was heated in a 16 mL capacity Teflon-lined reaction vessel at 433 K for 4 days. The reaction mixture was then cooled to room temperature over a period of 40 h. The product was collected by filtration, washed with water and air-dried.

### Refinement

H atoms bonded to C atoms were placed geometrically and refiined as riding atoms. The pyridyl N atoms were found from a difference Fourier maps and refined as riding, with N—H = 0.8600 Å, and the water H atoms were found from Fourier difference maps and refined with restraints for O—H distances (0.85 Å) with *U*_iso_(H) = 1.2*U*_eq_(O). The highest residual electron density was found at 0.88 Å from Er1 atom and the deepest hole at 0.91 Å from the Er1 atom.

### Crystal data

**Table:**

[CoEr_2_(C_14_H_8_N_2_O_5_)_4_(H_2_O)_4_]·4H_2_O	*Z* = 1
*M**_r_* = 1674.47	*F*(000) = 827
Triclinic, *P*1	*D*_x_ = 1.934 Mg m^−^^3^
Hall symbol: -P 1	Mo *K*α radiation, λ = 0.71073 Å
*a* = 10.0816 (9) Å	Cell parameters from 5230 reflections
*b* = 10.7844 (10) Å	θ = 2.3–28.1°
*c* = 13.7316 (12) Å	µ = 3.28 mm^−^^1^
α = 79.174 (1)°	*T* = 293 K
β = 78.771 (2)°	Block, pink
γ = 86.355 (2)°	0.20 × 0.18 × 0.10 mm
*V* = 1437.7 (2) Å^3^	

### Data collection

**Table:**

Bruker SMART CCD area-detector diffractometer	4979 independent reflections
Radiation source: fine-focus sealed tube	4649 reflections with *I* > 2σ(*I*)
graphite	*R*_int_ = 0.096
φ and ω scans	θ_max_ = 25.0°, θ_min_ = 1.9°
Absorption correction: multi-scan (*SADABS*; Bruker, 2000)	*h* = −5→12
*T*_min_ = 0.560, *T*_max_ = 0.735	*k* = −12→12
7172 measured reflections	*l* = −15→16

### Refinement

**Table:**

Refinement on *F*^2^	Primary atom site location: structure-invariant direct methods
Least-squares matrix: full	Secondary atom site location: difference Fourier map
*R*[*F*^2^ > 2σ(*F*^2^)] = 0.052	Hydrogen site location: inferred from neighbouring sites
*wR*(*F*^2^) = 0.136	H-atom parameters constrained
*S* = 1.02	*w* = 1/[σ^2^(*F*_o_^2^) + (0.1058*P*)^2^] where *P* = (*F*_o_^2^ + 2*F*_c_^2^)/3
4979 reflections	(Δ/σ)_max_ = 0.001
430 parameters	Δρ_max_ = 3.52 e Å^−^^3^
0 restraints	Δρ_min_ = −2.99 e Å^−^^3^

### Special details

**Table:**

Geometry. All e.s.d.'s (except the e.s.d. in the dihedral angle between two l.s. planes) are estimated using the full covariance matrix. The cell e.s.d.'s are taken into account individually in the estimation of e.s.d.'s in distances, angles and torsion angles; correlations between e.s.d.'s in cell parameters are only used when they are defined by crystal symmetry. An approximate (isotropic) treatment of cell e.s.d.'s is used for estimating e.s.d.'s involving l.s. planes.
Refinement. Refinement of *F*^2^ against ALL reflections. The weighted *R*-factor *wR* and goodness of fit *S* are based on *F*^2^, conventional *R*-factors *R* are based on *F*, with *F* set to zero for negative *F*^2^. The threshold expression of *F*^2^ > σ(*F*^2^) is used only for calculating *R*-factors(gt) *etc*. and is not relevant to the choice of reflections for refinement. *R*-factors based on *F*^2^ are statistically about twice as large as those based on *F*, and *R*- factors based on ALL data will be even larger.

### Fractional atomic coordinates and isotropic or equivalent isotropic displacement parameters (Å^2^)

**Table:**

	*x*	*y*	*z*	*U*_iso_*/*U*_eq_	
Co1	−0.5000	0.5000	1.0000	0.0223 (3)	
Er1	−0.18176 (2)	0.92224 (2)	0.796262 (18)	0.01355 (14)	
C1	−0.2010 (6)	0.5274 (6)	0.7723 (5)	0.0162 (12)	
C2	−0.1777 (7)	0.5835 (6)	0.6709 (5)	0.0203 (13)	
H2A	−0.1712	0.6707	0.6528	0.024*	
C3	−0.1640 (6)	0.5091 (6)	0.5959 (5)	0.0178 (13)	
C4	−0.1655 (6)	0.3777 (6)	0.6249 (5)	0.0187 (13)	
H4A	−0.1543	0.3266	0.5762	0.022*	
C5	−0.1833 (6)	0.3243 (6)	0.7250 (5)	0.0165 (12)	
C6	−0.2041 (6)	0.3972 (6)	0.8004 (5)	0.0187 (13)	
H6A	−0.2197	0.3594	0.8682	0.022*	
C7	−0.1869 (6)	0.1837 (6)	0.7518 (5)	0.0178 (13)	
C8	−0.2318 (7)	0.6074 (6)	0.8526 (5)	0.0205 (14)	
C9	−0.1651 (7)	0.5169 (7)	0.4170 (5)	0.0264 (15)	
C10	−0.1506 (7)	0.6000 (7)	0.3142 (5)	0.0239 (15)	
C11	−0.1432 (8)	0.5403 (8)	0.2318 (5)	0.0325 (17)	
H11A	−0.1416	0.4526	0.2406	0.039*	
C12	−0.1385 (9)	0.6116 (8)	0.1383 (5)	0.0356 (18)	
H12A	−0.1338	0.5698	0.0843	0.043*	
C13	−0.1448 (8)	0.7939 (8)	0.1996 (6)	0.0337 (17)	
H13A	−0.1442	0.8816	0.1888	0.040*	
C14	−0.1505 (8)	0.7294 (7)	0.2963 (5)	0.0284 (16)	
H14A	−0.1543	0.7730	0.3491	0.034*	
C15	0.2360 (7)	0.8871 (6)	0.6690 (5)	0.0198 (14)	
C16	0.2649 (6)	0.8586 (6)	0.5711 (5)	0.0188 (13)	
H16A	0.1946	0.8527	0.5375	0.023*	
C17	0.3943 (6)	0.8397 (6)	0.5257 (5)	0.0182 (13)	
C18	0.5006 (6)	0.8521 (6)	0.5738 (5)	0.0192 (13)	
H18A	0.5896	0.8420	0.5413	0.023*	
C19	0.4738 (6)	0.8796 (6)	0.6698 (5)	0.0191 (13)	
C20	0.3414 (6)	0.8968 (6)	0.7185 (5)	0.0199 (13)	
H20A	0.3237	0.9147	0.7835	0.024*	
C21	0.0908 (6)	0.9019 (6)	0.7170 (5)	0.0195 (13)	
C22	0.5927 (6)	0.8916 (6)	0.7177 (5)	0.0181 (13)	
C23	0.5058 (6)	0.7065 (7)	0.4098 (5)	0.0211 (14)	
C24	0.5094 (6)	0.6636 (7)	0.3100 (5)	0.0226 (14)	
C25	0.5104 (7)	0.5355 (7)	0.3108 (5)	0.0258 (15)	
H25A	0.5077	0.4783	0.3709	0.031*	
C26	0.5155 (7)	0.4933 (7)	0.2211 (5)	0.0274 (15)	
H26A	0.5150	0.4068	0.2225	0.033*	
C27	0.5258 (8)	0.6949 (7)	0.1321 (5)	0.0300 (16)	
H27A	0.5354	0.7497	0.0703	0.036*	
C28	0.5172 (7)	0.7460 (7)	0.2184 (5)	0.0262 (15)	
H28A	0.5167	0.8330	0.2151	0.031*	
N1	−0.1457 (5)	0.5694 (5)	0.4954 (4)	0.0197 (11)	
H1	−0.1199	0.6463	0.4819	0.024*	
N2	−0.1400 (7)	0.7376 (7)	0.1191 (5)	0.0352 (15)	
N3	0.4213 (5)	0.8043 (5)	0.4280 (4)	0.0199 (12)	
H3	0.3831	0.8460	0.3806	0.024*	
N4	0.5212 (6)	0.5710 (6)	0.1323 (4)	0.0243 (13)	
O1	−0.3059 (5)	0.5656 (5)	0.9346 (3)	0.0292 (11)	
O2	−0.1793 (5)	0.7147 (4)	0.8335 (4)	0.0280 (11)	
O3	−0.1703 (6)	0.1271 (4)	0.8379 (3)	0.0325 (12)	
O4	−0.2075 (6)	0.1200 (5)	0.6898 (4)	0.0331 (12)	
O5	−0.1926 (8)	0.4070 (5)	0.4251 (4)	0.0521 (18)	
O6	0.0561 (5)	0.9015 (5)	0.8108 (4)	0.0279 (12)	
O7	0.0016 (5)	0.9159 (6)	0.6644 (3)	0.0356 (13)	
O8	0.5758 (5)	0.9079 (5)	0.8090 (4)	0.0285 (12)	
O9	0.7095 (4)	0.8844 (4)	0.6686 (3)	0.0214 (10)	
O10	0.5752 (5)	0.6497 (6)	0.4668 (4)	0.0357 (13)	
O1W	−0.4267 (6)	0.3145 (5)	1.0665 (4)	0.0339 (12)	
H1WB	−0.3591	0.2868	1.0285	0.041*	
H1WA	−0.4880	0.2603	1.0843	0.041*	
O2W	−0.2229 (6)	0.9033 (5)	0.9693 (4)	0.0335 (12)	
H2WB	−0.1908	0.9650	0.9863	0.040*	
H2WC	−0.1880	0.8351	0.9967	0.040*	
O3W	0.9154 (8)	0.8418 (6)	0.4834 (4)	0.0568 (19)	
H3WA	0.9711	0.8601	0.5171	0.068*	
H3WB	0.8369	0.8457	0.5189	0.068*	
O4W	0.8025 (7)	0.1615 (8)	0.0328 (5)	0.066 (2)	
H4WA	0.8294	0.1409	−0.0248	0.079*	
H4WB	0.8595	0.1331	0.0702	0.079*	

### Atomic displacement parameters (Å^2^)

**Table:**

	*U*^11^	*U*^22^	*U*^33^	*U*^12^	*U*^13^	*U*^23^
Co1	0.0270 (7)	0.0208 (7)	0.0212 (6)	−0.0031 (5)	−0.0058 (5)	−0.0071 (5)
Er1	0.0144 (2)	0.0097 (2)	0.0188 (2)	0.00035 (12)	−0.00753 (12)	−0.00390 (12)
C1	0.017 (3)	0.011 (3)	0.021 (3)	−0.001 (2)	−0.005 (2)	−0.004 (2)
C2	0.023 (3)	0.012 (3)	0.026 (3)	0.000 (3)	−0.004 (3)	−0.003 (3)
C3	0.016 (3)	0.015 (3)	0.023 (3)	0.001 (2)	−0.005 (2)	−0.003 (2)
C4	0.017 (3)	0.016 (3)	0.026 (3)	−0.002 (2)	−0.006 (3)	−0.007 (3)
C5	0.016 (3)	0.012 (3)	0.023 (3)	0.002 (2)	−0.007 (2)	−0.004 (2)
C6	0.017 (3)	0.016 (3)	0.023 (3)	−0.001 (3)	−0.007 (3)	−0.001 (3)
C7	0.014 (3)	0.018 (3)	0.021 (3)	0.000 (3)	−0.006 (2)	−0.002 (3)
C8	0.021 (3)	0.017 (3)	0.027 (3)	0.008 (3)	−0.013 (3)	−0.006 (3)
C9	0.031 (4)	0.024 (4)	0.026 (3)	0.000 (3)	−0.012 (3)	−0.005 (3)
C10	0.018 (3)	0.029 (4)	0.028 (4)	−0.001 (3)	−0.010 (3)	−0.006 (3)
C11	0.041 (4)	0.029 (4)	0.031 (4)	0.002 (3)	−0.011 (3)	−0.009 (3)
C12	0.045 (5)	0.042 (5)	0.022 (4)	0.001 (4)	−0.008 (3)	−0.011 (3)
C13	0.040 (4)	0.028 (4)	0.035 (4)	−0.007 (3)	−0.016 (3)	0.002 (3)
C14	0.032 (4)	0.030 (4)	0.026 (4)	−0.003 (3)	−0.014 (3)	−0.002 (3)
C15	0.017 (3)	0.016 (3)	0.028 (3)	0.001 (3)	−0.008 (3)	−0.006 (3)
C16	0.016 (3)	0.020 (3)	0.024 (3)	0.002 (3)	−0.012 (2)	−0.005 (3)
C17	0.022 (3)	0.014 (3)	0.022 (3)	0.000 (3)	−0.010 (3)	−0.006 (2)
C18	0.014 (3)	0.019 (3)	0.025 (3)	0.000 (2)	−0.007 (3)	−0.003 (3)
C19	0.013 (3)	0.019 (3)	0.027 (3)	−0.003 (3)	−0.008 (3)	−0.003 (3)
C20	0.022 (3)	0.023 (3)	0.019 (3)	0.002 (3)	−0.009 (3)	−0.011 (3)
C21	0.020 (3)	0.013 (3)	0.028 (3)	−0.003 (2)	−0.009 (3)	−0.006 (3)
C22	0.018 (3)	0.011 (3)	0.029 (3)	−0.006 (2)	−0.007 (3)	−0.008 (3)
C23	0.015 (3)	0.031 (4)	0.019 (3)	−0.001 (3)	−0.005 (3)	−0.006 (3)
C24	0.009 (3)	0.035 (4)	0.026 (3)	0.002 (3)	−0.003 (2)	−0.011 (3)
C25	0.031 (4)	0.030 (4)	0.020 (3)	0.002 (3)	−0.009 (3)	−0.009 (3)
C26	0.036 (4)	0.027 (4)	0.024 (3)	0.004 (3)	−0.012 (3)	−0.013 (3)
C27	0.037 (4)	0.031 (4)	0.022 (3)	−0.007 (3)	−0.006 (3)	−0.002 (3)
C28	0.026 (4)	0.029 (4)	0.028 (3)	−0.003 (3)	−0.006 (3)	−0.013 (3)
N1	0.025 (3)	0.013 (3)	0.022 (3)	0.001 (2)	−0.009 (2)	0.000 (2)
N2	0.040 (4)	0.040 (4)	0.027 (3)	−0.004 (3)	−0.016 (3)	0.001 (3)
N3	0.022 (3)	0.026 (3)	0.015 (3)	0.002 (2)	−0.010 (2)	−0.006 (2)
N4	0.021 (3)	0.029 (3)	0.026 (3)	−0.003 (2)	−0.003 (2)	−0.013 (3)
O1	0.030 (3)	0.032 (3)	0.025 (2)	−0.009 (2)	0.001 (2)	−0.009 (2)
O2	0.043 (3)	0.009 (2)	0.033 (3)	−0.005 (2)	−0.010 (2)	−0.0046 (19)
O3	0.069 (4)	0.011 (2)	0.023 (2)	−0.004 (2)	−0.020 (2)	−0.0013 (19)
O4	0.062 (4)	0.017 (3)	0.029 (3)	−0.002 (2)	−0.025 (2)	−0.007 (2)
O5	0.106 (6)	0.021 (3)	0.035 (3)	−0.020 (3)	−0.028 (3)	0.002 (2)
O6	0.019 (2)	0.045 (3)	0.024 (2)	−0.001 (2)	−0.0048 (19)	−0.015 (2)
O7	0.017 (2)	0.066 (4)	0.024 (2)	0.005 (2)	−0.007 (2)	−0.005 (2)
O8	0.022 (3)	0.046 (3)	0.025 (2)	−0.002 (2)	−0.009 (2)	−0.017 (2)
O9	0.012 (2)	0.026 (3)	0.029 (2)	0.0022 (18)	−0.0079 (18)	−0.010 (2)
O10	0.034 (3)	0.046 (3)	0.035 (3)	0.018 (3)	−0.021 (2)	−0.020 (2)
O1W	0.040 (3)	0.029 (3)	0.033 (3)	0.000 (2)	−0.007 (2)	−0.007 (2)
O2W	0.054 (3)	0.021 (3)	0.026 (2)	0.002 (2)	−0.016 (2)	0.000 (2)
O3W	0.104 (6)	0.038 (4)	0.032 (3)	−0.018 (4)	−0.013 (3)	−0.012 (3)
O4W	0.053 (4)	0.111 (7)	0.034 (3)	0.023 (4)	−0.015 (3)	−0.014 (4)

### Geometric parameters (Å, °)

**Table:**

Co1—O1^i^	2.095 (5)	C15—C16	1.406 (9)
Co1—O1	2.095 (5)	C15—C21	1.495 (9)
Co1—N4^ii^	2.152 (5)	C16—C17	1.353 (9)
Co1—N4^iii^	2.152 (5)	C16—H16A	0.9300
Co1—O1W	2.184 (5)	C17—C18	1.390 (9)
Co1—O1W^i^	2.184 (5)	C17—N3	1.434 (8)
Er1—O2	2.200 (5)	C18—C19	1.377 (9)
Er1—O2W	2.303 (5)	C18—H18A	0.9300
Er1—O7	2.330 (5)	C19—C20	1.389 (9)
Er1—O9^iv^	2.348 (4)	C19—C22	1.498 (9)
Er1—O4^v^	2.379 (5)	C20—H20A	0.9300
Er1—O3^v^	2.398 (5)	C21—O7	1.245 (8)
Er1—O8^iv^	2.429 (5)	C21—O6	1.266 (8)
Er1—O6	2.438 (5)	C22—O9	1.243 (8)
C1—C6	1.385 (9)	C22—O8	1.277 (8)
C1—C2	1.390 (9)	C23—O10	1.207 (8)
C1—C8	1.500 (8)	C23—N3	1.342 (9)
C2—C3	1.402 (9)	C23—C24	1.521 (9)
C2—H2A	0.9300	C24—C25	1.379 (10)
C3—C4	1.398 (9)	C24—C28	1.389 (10)
C3—N1	1.393 (8)	C25—C26	1.382 (9)
C4—C5	1.368 (9)	C25—H25A	0.9300
C4—H4A	0.9300	C26—N4	1.336 (10)
C5—C6	1.392 (9)	C26—H26A	0.9300
C5—C7	1.492 (9)	C27—N4	1.339 (10)
C6—H6A	0.9300	C27—C28	1.384 (10)
C7—O4	1.244 (8)	C27—H27A	0.9300
C7—O3	1.261 (8)	C28—H28A	0.9300
C8—O1	1.247 (8)	N1—H1	0.8600
C8—O2	1.263 (8)	N3—H3	0.8600
C9—O5	1.214 (9)	N4—Co1^vi^	2.152 (5)
C9—N1	1.358 (8)	O3—Er1^vii^	2.398 (5)
C9—C10	1.509 (10)	O4—Er1^vii^	2.379 (5)
C10—C14	1.371 (11)	O8—Er1^viii^	2.429 (5)
C10—C11	1.391 (10)	O9—Er1^viii^	2.348 (4)
C11—C12	1.362 (11)	O1W—H1WB	0.8488
C11—H11A	0.9300	O1W—H1WA	0.8469
C12—N2	1.334 (11)	O2W—H2WB	0.8474
C12—H12A	0.9300	O2W—H2WC	0.8489
C13—N2	1.349 (10)	O3W—H3WA	0.8477
C13—C14	1.370 (10)	O3W—H3WB	0.8482
C13—H13A	0.9300	O4W—H4WA	0.8494
C14—H14A	0.9300	O4W—H4WB	0.8487
C15—C20	1.387 (9)		
			
O1^i^—Co1—O1	180.0	O5—C9—C10	118.5 (6)
O1^i^—Co1—N4^ii^	87.4 (2)	N1—C9—C10	117.9 (6)
O1—Co1—N4^ii^	92.6 (2)	C14—C10—C11	117.6 (7)
O1^i^—Co1—N4^iii^	92.6 (2)	C14—C10—C9	125.2 (6)
O1—Co1—N4^iii^	87.4 (2)	C11—C10—C9	117.1 (7)
N4^ii^—Co1—N4^iii^	180.000 (1)	C12—C11—C10	119.3 (7)
O1^i^—Co1—O1W	85.9 (2)	C12—C11—H11A	120.4
O1—Co1—O1W	94.1 (2)	C10—C11—H11A	120.4
N4^ii^—Co1—O1W	90.5 (2)	N2—C12—C11	124.2 (7)
N4^iii^—Co1—O1W	89.5 (2)	N2—C12—H12A	117.9
O1^i^—Co1—O1W^i^	94.1 (2)	C11—C12—H12A	117.9
O1—Co1—O1W^i^	85.9 (2)	N2—C13—C14	123.9 (7)
N4^ii^—Co1—O1W^i^	89.5 (2)	N2—C13—H13A	118.1
N4^iii^—Co1—O1W^i^	90.5 (2)	C14—C13—H13A	118.1
O1W—Co1—O1W^i^	180.000 (1)	C13—C14—C10	119.3 (7)
O2—Er1—O2W	82.38 (18)	C13—C14—H14A	120.3
O2—Er1—O7	90.7 (2)	C10—C14—H14A	120.3
O2W—Er1—O7	138.81 (18)	C20—C15—C16	119.5 (6)
O2—Er1—O9^iv^	81.92 (18)	C20—C15—C21	122.5 (6)
O2W—Er1—O9^iv^	139.01 (18)	C16—C15—C21	118.0 (6)
O7—Er1—O9^iv^	78.93 (16)	C17—C16—C15	120.4 (6)
O2—Er1—O4^v^	153.92 (18)	C17—C16—H16A	119.8
O2W—Er1—O4^v^	121.33 (17)	C15—C16—H16A	119.8
O7—Er1—O4^v^	78.2 (2)	C16—C17—C18	120.4 (6)
O9^iv^—Er1—O4^v^	72.87 (16)	C16—C17—N3	119.6 (6)
O2—Er1—O3^v^	152.42 (18)	C18—C17—N3	120.0 (6)
O2W—Er1—O3^v^	71.32 (17)	C19—C18—C17	119.7 (6)
O7—Er1—O3^v^	103.5 (2)	C19—C18—H18A	120.1
O9^iv^—Er1—O3^v^	123.62 (17)	C17—C18—H18A	120.1
O4^v^—Er1—O3^v^	53.59 (16)	C18—C19—C20	120.6 (6)
O2—Er1—O8^iv^	85.79 (19)	C18—C19—C22	117.2 (6)
O2W—Er1—O8^iv^	87.16 (18)	C20—C19—C22	122.2 (6)
O7—Er1—O8^iv^	132.96 (16)	C19—C20—C15	119.3 (6)
O9^iv^—Er1—O8^iv^	54.11 (15)	C19—C20—H20A	120.4
O4^v^—Er1—O8^iv^	84.85 (19)	C15—C20—H20A	120.4
O3^v^—Er1—O8^iv^	100.68 (19)	O7—C21—O6	118.8 (6)
O2—Er1—O6	85.14 (19)	O7—C21—C15	119.9 (6)
O2W—Er1—O6	85.04 (18)	O6—C21—C15	121.3 (6)
O7—Er1—O6	53.86 (16)	O9—C22—O8	119.3 (6)
O9^iv^—Er1—O6	130.75 (15)	O9—C22—C19	120.0 (6)
O4^v^—Er1—O6	106.15 (19)	O8—C22—C19	120.8 (6)
O3^v^—Er1—O6	84.49 (19)	O9—C22—Er1^viii^	57.8 (3)
O8^iv^—Er1—O6	168.74 (18)	O8—C22—Er1^viii^	61.5 (3)
O2—Er1—C22^iv^	83.91 (19)	C19—C22—Er1^viii^	176.9 (4)
O2W—Er1—C22^iv^	113.99 (19)	O10—C23—N3	125.3 (6)
O7—Er1—C22^iv^	105.45 (18)	O10—C23—C24	118.6 (6)
O9^iv^—Er1—C22^iv^	26.61 (17)	N3—C23—C24	116.1 (6)
O4^v^—Er1—C22^iv^	76.62 (18)	C25—C24—C28	118.8 (6)
O3^v^—Er1—C22^iv^	113.96 (19)	C25—C24—C23	117.6 (6)
O8^iv^—Er1—C22^iv^	27.52 (17)	C28—C24—C23	123.5 (6)
O6—Er1—C22^iv^	156.44 (18)	C26—C25—C24	119.1 (7)
O2—Er1—C7^v^	179.27 (18)	C26—C25—H25A	120.4
O2W—Er1—C7^v^	96.94 (18)	C24—C25—H25A	120.4
O7—Er1—C7^v^	90.0 (2)	N4—C26—C25	123.0 (7)
O9^iv^—Er1—C7^v^	98.43 (17)	N4—C26—H26A	118.5
O4^v^—Er1—C7^v^	26.59 (17)	C25—C26—H26A	118.5
O3^v^—Er1—C7^v^	27.03 (17)	N4—C27—C28	123.8 (7)
O8^iv^—Er1—C7^v^	93.89 (19)	N4—C27—H27A	118.1
O6—Er1—C7^v^	95.10 (19)	C28—C27—H27A	118.1
C22^iv^—Er1—C7^v^	96.11 (18)	C24—C28—C27	118.0 (7)
C6—C1—C2	120.6 (6)	C24—C28—H28A	121.0
C6—C1—C8	119.0 (6)	C27—C28—H28A	121.0
C2—C1—C8	120.3 (6)	C9—N1—C3	125.4 (6)
C1—C2—C3	120.3 (6)	C9—N1—H1	117.3
C1—C2—H2A	119.9	C3—N1—H1	117.3
C3—C2—H2A	119.9	C12—N2—C13	115.7 (6)
C4—C3—N1	122.8 (6)	C23—N3—C17	120.5 (5)
C4—C3—C2	118.7 (6)	C23—N3—H3	119.7
N1—C3—C2	118.4 (6)	C17—N3—H3	119.7
C5—C4—C3	120.0 (6)	C26—N4—C27	117.2 (6)
C5—C4—H4A	120.0	C26—N4—Co1^vi^	120.6 (5)
C3—C4—H4A	120.0	C27—N4—Co1^vi^	121.8 (5)
C4—C5—C6	121.9 (6)	C8—O1—Co1	143.4 (4)
C4—C5—C7	117.8 (6)	C8—O2—Er1	154.6 (5)
C6—C5—C7	120.3 (6)	C7—O3—Er1^vii^	93.2 (4)
C1—C6—C5	118.4 (6)	C7—O4—Er1^vii^	94.6 (4)
C1—C6—H6A	120.8	C21—O6—Er1	90.8 (4)
C5—C6—H6A	120.8	C21—O7—Er1	96.5 (4)
O4—C7—O3	118.6 (6)	C22—O8—Er1^viii^	90.9 (4)
O4—C7—C5	120.5 (6)	C22—O9—Er1^viii^	95.6 (4)
O3—C7—C5	120.9 (6)	Co1—O1W—H1WB	112.7
O4—C7—Er1^vii^	58.9 (3)	Co1—O1W—H1WA	112.9
O3—C7—Er1^vii^	59.8 (3)	H1WB—O1W—H1WA	110.2
C5—C7—Er1^vii^	177.4 (5)	Er1—O2W—H2WB	110.5
O1—C8—O2	123.2 (6)	Er1—O2W—H2WC	111.1
O1—C8—C1	119.2 (6)	H2WB—O2W—H2WC	109.0
O2—C8—C1	117.6 (6)	H3WA—O3W—H3WB	107.4
O5—C9—N1	123.6 (6)	H4WA—O4W—H4WB	108.9
			
C6—C1—C2—C3	3.3 (10)	O5—C9—N1—C3	−5.1 (12)
C8—C1—C2—C3	−172.9 (6)	C10—C9—N1—C3	175.4 (6)
C1—C2—C3—C4	−4.1 (9)	C4—C3—N1—C9	19.7 (10)
C1—C2—C3—N1	177.7 (6)	C2—C3—N1—C9	−162.1 (6)
N1—C3—C4—C5	179.7 (6)	C11—C12—N2—C13	0.9 (12)
C2—C3—C4—C5	1.6 (9)	C14—C13—N2—C12	−1.2 (12)
C3—C4—C5—C6	1.8 (10)	O10—C23—N3—C17	−8.0 (11)
C3—C4—C5—C7	178.8 (6)	C24—C23—N3—C17	170.3 (6)
C2—C1—C6—C5	0.0 (9)	C16—C17—N3—C23	−131.1 (7)
C8—C1—C6—C5	176.2 (6)	C18—C17—N3—C23	47.8 (9)
C4—C5—C6—C1	−2.6 (10)	C25—C26—N4—C27	1.9 (11)
C7—C5—C6—C1	−179.5 (6)	C25—C26—N4—Co1^vi^	−171.1 (6)
C4—C5—C7—O4	−17.8 (9)	C28—C27—N4—C26	−3.9 (11)
C6—C5—C7—O4	159.2 (6)	C28—C27—N4—Co1^vi^	169.1 (6)
C4—C5—C7—O3	162.8 (6)	O2—C8—O1—Co1	120.6 (7)
C6—C5—C7—O3	−20.2 (10)	C1—C8—O1—Co1	−60.1 (10)
C6—C1—C8—O1	−26.8 (9)	N4^ii^—Co1—O1—C8	29.9 (8)
C2—C1—C8—O1	149.4 (6)	N4^iii^—Co1—O1—C8	−150.1 (8)
C6—C1—C8—O2	152.5 (6)	O1W—Co1—O1—C8	120.6 (8)
C2—C1—C8—O2	−31.2 (9)	O1W^i^—Co1—O1—C8	−59.4 (8)
O5—C9—C10—C14	164.4 (8)	O1—C8—O2—Er1	−74.1 (13)
N1—C9—C10—C14	−16.0 (11)	C1—C8—O2—Er1	106.6 (10)
O5—C9—C10—C11	−12.4 (11)	O2W—Er1—O2—C8	91.1 (11)
N1—C9—C10—C11	167.2 (6)	O7—Er1—O2—C8	−129.7 (11)
C14—C10—C11—C12	−1.0 (11)	O9^iv^—Er1—O2—C8	−51.0 (11)
C9—C10—C11—C12	176.0 (7)	O4^v^—Er1—O2—C8	−65.8 (12)
C10—C11—C12—N2	0.2 (13)	O3^v^—Er1—O2—C8	108.5 (11)
N2—C13—C14—C10	0.4 (12)	O8^iv^—Er1—O2—C8	3.4 (11)
C11—C10—C14—C13	0.7 (11)	O6—Er1—O2—C8	176.7 (11)
C9—C10—C14—C13	−176.0 (7)	C22^iv^—Er1—O2—C8	−24.2 (11)
C20—C15—C16—C17	0.6 (10)	O4—C7—O3—Er1^vii^	3.6 (7)
C21—C15—C16—C17	−177.6 (6)	C5—C7—O3—Er1^vii^	−177.1 (5)
C15—C16—C17—C18	−2.1 (10)	O3—C7—O4—Er1^vii^	−3.6 (7)
C15—C16—C17—N3	176.8 (6)	C5—C7—O4—Er1^vii^	177.0 (5)
C16—C17—C18—C19	2.3 (10)	O7—C21—O6—Er1	2.5 (7)
N3—C17—C18—C19	−176.6 (6)	C15—C21—O6—Er1	−178.0 (6)
C17—C18—C19—C20	−1.0 (10)	O2—Er1—O6—C21	92.9 (4)
C17—C18—C19—C22	179.9 (6)	O2W—Er1—O6—C21	175.7 (4)
C18—C19—C20—C15	−0.5 (10)	O7—Er1—O6—C21	−1.5 (4)
C22—C19—C20—C15	178.6 (6)	O9^iv^—Er1—O6—C21	17.9 (5)
C16—C15—C20—C19	0.7 (10)	O4^v^—Er1—O6—C21	−63.1 (4)
C21—C15—C20—C19	178.8 (6)	O3^v^—Er1—O6—C21	−112.7 (4)
C20—C15—C21—O7	166.6 (6)	O8^iv^—Er1—O6—C21	129.4 (7)
C16—C15—C21—O7	−15.3 (10)	C22^iv^—Er1—O6—C21	30.4 (7)
C20—C15—C21—O6	−12.9 (10)	C7^v^—Er1—O6—C21	−87.8 (4)
C16—C15—C21—O6	165.2 (6)	O6—C21—O7—Er1	−2.7 (7)
C18—C19—C22—O9	4.3 (9)	C15—C21—O7—Er1	177.8 (5)
C20—C19—C22—O9	−174.8 (6)	O2—Er1—O7—C21	−82.0 (4)
C18—C19—C22—O8	−174.9 (6)	O2W—Er1—O7—C21	−2.8 (6)
C20—C19—C22—O8	6.0 (10)	O9^iv^—Er1—O7—C21	−163.6 (5)
O10—C23—C24—C25	41.6 (9)	O4^v^—Er1—O7—C21	121.8 (4)
N3—C23—C24—C25	−136.8 (7)	O3^v^—Er1—O7—C21	74.1 (4)
O10—C23—C24—C28	−135.7 (7)	O8^iv^—Er1—O7—C21	−166.9 (4)
N3—C23—C24—C28	45.9 (9)	O6—Er1—O7—C21	1.5 (4)
C28—C24—C25—C26	−1.7 (10)	C22^iv^—Er1—O7—C21	−165.9 (4)
C23—C24—C25—C26	−179.1 (6)	C7^v^—Er1—O7—C21	97.8 (4)
C24—C25—C26—N4	0.8 (11)	O9—C22—O8—Er1^viii^	3.2 (6)
C25—C24—C28—C27	−0.1 (10)	C19—C22—O8—Er1^viii^	−177.6 (5)
C23—C24—C28—C27	177.2 (6)	O8—C22—O9—Er1^viii^	−3.3 (7)
N4—C27—C28—C24	3.0 (11)	C19—C22—O9—Er1^viii^	177.4 (5)

Symmetry codes: (i) −*x*−1, −*y*+1, −*z*+2; (ii) −*x*, −*y*+1, −*z*+1; (iii) *x*−1, *y*, *z*+1; (iv) *x*−1, *y*, *z*; (v) *x*, *y*+1, *z*; (vi) *x*+1, *y*, *z*−1; (vii) *x*, *y*−1, *z*; (viii) *x*+1, *y*, *z*.

### Hydrogen-bond geometry (Å, °)

**Table:**

*D*—H···*A*	*D*—H	H···*A*	*D*···*A*	*D*—H···*A*
N1—H1···O3W^iv^	0.86	2.16	3.007 (8)	166.
O1W—H1WB···O4W^iii^	0.85	2.05	2.769 (9)	142.
O1W—H1WA···O8^ix^	0.85	2.22	2.975 (8)	149.
O2W—H2WB···O4W^x^	0.85	2.32	3.109 (10)	156.
O2W—H2WC···N2^xi^	0.85	1.94	2.687 (8)	147.
O3W—H3WA···O7^viii^	0.85	2.30	3.041 (8)	147.
O3W—H3WB···O9	0.85	2.30	3.042 (8)	147.
O4W—H4WA···O3^vi^	0.85	1.92	2.729 (8)	159.
O4W—H4WB···O6^xii^	0.85	1.95	2.762 (8)	159.

Symmetry codes: (iv) *x*−1, *y*, *z*; (iii) *x*−1, *y*, *z*+1; (ix) −*x*, −*y*+1, −*z*+2; (x) *x*−1, *y*+1, *z*+1; (xi) *x*, *y*, *z*+1; (viii) *x*+1, *y*, *z*; (vi) *x*+1, *y*, *z*−1; (xii) −*x*+1, −*y*+1, −*z*+1.
